# Biotransformation and tissue distribution of protopine and allocryptopine and effects of Plume Poppy Total Alkaloid on liver drug-metabolizing enzymes

**DOI:** 10.1038/s41598-017-18816-7

**Published:** 2018-01-11

**Authors:** Ya-Jun Huang, Pi Cheng, Zhuo-Yi Zhang, Shi-Jie Tian, Zhi-Liang Sun, Jian-Guo Zeng, Zhao-Ying Liu

**Affiliations:** 1 National and Local Union Engineering Research Center for the Veterinary Herbal Medicine Resources and Initiative, Hunan Agricultural University, Changsha, 410128 China; 2 Hunan Engineering Research Center of Veterinary Drug, College of Veterinary Medicine, Hunan Agricultural University, Changsha, 410128 China; 3 Hunan Key Laboratory of Traditional Chinese Veterinary Medicine, Hunan Agricultural University, Changsha, 410128 China

## Abstract

In this study, the biotransformation in the plasma, urine and feces of rats following oral administration of protopine (PRO) and allocryptopine (ALL)were explored using HPLC-QqTOF MS. An HPLC-MS/MS method for the determination of tissues was developed and applied to the tissue distribution study in rats following intragastric administration of Plume Poppy Total Alkaloid for 3 weeks. A total of ten PRO metabolites and ten ALL metabolites were characterized in rats *in vivo*. Among these metabolites, six PRO metabolites and five ALL metabolites were reported for the first time. The predicated metabolic pathways including ring cleavage, demethylation following ring cleavage, and glucuronidation were proposed. The low-concentration residue of PRO and ALL in various tissues was detected at 24 h and 48 h after dosing, which indicated that both compounds could be widely distributed in tissues and exist as low levels of residue. The activities of erythromycin N-demethylase, aminopyrine N-demethylase and NAD (P)H quinone oxidoreductase in female rats can be induced post-dose, but these activities were inhibited in male rats. The proposed biotransformation and residues of PRO and ALL and their effects on enzymes may provide a basis for clarifying the metabolism and interpreting pharmacokinetics.

## Introduction

The isoquinoline alkaloids protopine (PRO) and allocryptopine (ALL) are found primarily in the plant families *Fumariaceae*, *Papaveraceae*, *Berberidaceae* and *Ranunculaceae*^1–5^. Both are also biologically active substances in human and veterinary phytopreparations from medicinal plants such as *Chelidonium majus* and *Macleaya cordata*^6^. PRO and ALL have many demonstrated biological activities, such as anti-thrombotic, anti-inflammatory^7^, anti-parasitic activity^8^, antimicrobial activity^9^, as well as hepatoprotective effects in animal models^10^. These biological activities of PRO and ALL are associated with its ability to inhibit K (ATP) channels^11–13^. Thus, the both alkaloids and associated plants have attracted increasing attention from pharmacologists due to their multiple biological effects.

Pharmacokinetic studies have indicated that PRO and ALL are rapidly absorbed after oral administration^14,15^. Other pharmacokinetics studies of PRO also showed that the absolute bioavailability of PRO was apparently low and PRO underwent a rapid, wide distribution into tissues. The study also found that only <1.0% of excreted PRO was unconverted, which indicated that PRO is mainly excreted as its metabolite^16^. Previously, two dioxolo-ring-opened PRO metabolites of the 2, 3-methylenedioxy group were found in the urine of rats using gas chromatography (GC)-mass spectrometry (MS)^17^. One demethylenated metabolite, two dioxolo-ring opened metabolites and three tetrahydroprotoberberines metabolites of PRO were identified in urine via GC-MS^18^. The demethylenated metabolites of the 2, 3-methylenedioxy group and 9, 10-methylenedioxy group were also found in rat plasma after oral administration of the Yuanhu Zhitong prescription extract^19^. Only one report studied ALL metabolism after oral administration of the active Xiang-Fu-Si-Wu Decoction fraction^20^. However, the *in vivo* biotransformation of PRO and ALL have not been systematically illustrated. The detailed structural elucidation of ALL and PRO metabolites has been little reported. Therefore, it is crucial to obtain more detailed information about the biotransformation and tissue distribution of these two compounds *in vivo*.

In our recent research^21^, a total five PRO metabolites (PR1-PR5) and eight ALL metabolites (AL1-AL8) were detected and characterized in rat liver S9 using rapid, accurate high-performance liquid chromatography/quadrupole-time-of-flight mass spectrometry (HPLC-QqTOF MS). Two PRO metabolites and seven ALL metabolites were identified for the first time. However, our *in vitro* findings for new metabolites of PRO and ALL correlate with the *in vivo* metabolism and require further studies. Currently, PRO and ALL in phytopreparations from the medicinal plants could be proven substrates of drug-metabolizing enzymes and might lead to herb-drug interactions via the induction or inhibition of the enzymes. Inhibition studies on rat liver microsomes indicated that CYP2D1 and CYP2C11 were primarily involved in demethylenation of PRO, while CYP1A2 and CYP3A2 showed only minor contributions^22^. PRO and ALL have been also shown to inhibit several human CYP enzymes^23^. However, CYP1A mRNA levels induced by both alkaloids in both HepG2 cells and human hepatocytes did not result in elevated CYP1A protein or activity levels^24^. Recently, significant activation of the pregnane X receptor was observed with ALL in HepG2 cells^25^. However, the effects of PRO and ALL on drug-metabolizing enzymes *in vivo* have not been assessed to date.

Therefore, the first aim of the present study was to explore in detail the *in vivo* metabolism of PRO and ALL in male and female rats. This study determined the biotransformation in plasma, urine and feces following a single oral administration of PRO and ALL. The second aim of this study was to investigate the tissue metabolites residues and the effects on drug-metabolizing enzymes following intragastric administered with Plume Poppy Total Alkoloid (containing 15% ALL and 35% PRO) derived from *Macleaya cordata* for 3 weeks. To achieve this goal, we developed an HPLC-QqTOF MS method to characterize the metabolites formed by accurate mass measurements of MS and MS^2^ spectra and a high-performance liquid chromatography/triple-quadrupole mass (HPLC-MS/MS) method to quantify ALL and PRO in rat liver, kidney, spleen, lung and heart.

## Results

### Biotransformation of PRO in plasma, urine and feces

Table 1 lists metabolites of PRO detected in the plasma, urine and feces of male and female rats after dosing. No parent drug or PRO metabolite wasdetected in plasma at 3 h after intragastric administration of PRO in female and male rats. However, the parent drug and six PRO metabolites, PR6, PR7, PR8, PR9, PR10, and PR11, were detected in female rat urine at 0–24 h after intragastric administration of PRO. The accurate extracted ion chromatograms (EIC) of female rat urine between 0 h and 24 h after oral administration of a single dose of PRO are shown in Fig. S1. (See supplemental material). In addition to the unchanged parent drug, six metabolites observed in female rat urine were also observed in male rat urine at 0–24 h. The results indicated that PRO was quickly absorbed from the gastrointestinal tract and excreted rapidly. As seen in Table 1, no parent drug was found in female and male rat feces, but two PRO metabolites, PR2and PR6, were detected in rat feces.

**Table 1:** Summary of PRO and ALL metabolites detected in plasma, urine, feces, tissues and cecal contents of rats.

Compound	Plasma		Urine		Feces		Heart		Liver		Spleen		Lung			Kidney		Cecal contents	
	Female	male	Female	male	Female	male	24 h	48 h	24 h	48 h	24 h	48 h	24 h	48 h		24 h	48 h	24 h	48 h
PRO	ND	ND	√	ND	ND	ND	ND	ND	ND	√	ND	ND	ND		ND	ND	ND	ND	ND
PR1	ND	ND	ND	ND	ND	ND	ND	ND	ND	ND	ND	ND	ND		ND	ND	ND	ND	√
PR2	ND	ND	ND	ND	√	√	ND	ND	ND	ND	ND	ND	ND		ND	ND	ND	√	√
PR3	ND	ND	ND	ND	ND	ND	ND	ND	ND	ND	ND	ND	ND		ND	ND	ND	√	√
PR6	ND	ND	√	√	√	√	ND	ND	ND	ND	ND	ND	ND		ND	ND	ND	ND	√
PR7	ND	ND	√	√	ND	ND	ND	ND	ND	ND	ND	ND	ND		ND	ND	ND	ND	ND
PR8	ND	ND	√	√	ND	ND	ND	ND	ND	ND	ND	ND	ND		ND	ND	ND	ND	ND
PR9	ND	ND	√	√	ND	ND	ND	ND	ND	ND	ND	ND	ND		ND	ND	ND	ND	ND
PR10	ND	ND	√	√	ND	ND	ND	ND	ND	ND	ND	ND	ND		ND	ND	ND	ND	ND
PR11	ND	ND	√	√	ND	ND	ND	ND	ND	ND	ND	ND	ND		ND	ND	ND	ND	ND
PR12	ND	ND	ND	√	ND	ND	ND	ND	ND	ND	ND	ND	ND		ND	ND	ND	ND	ND
ALL	ND	ND	√	√	√	√	ND	ND	ND	ND	ND	ND	ND		ND	ND	ND	ND	ND
AL2	ND	ND	√	√	ND	ND	ND	ND	ND	ND	ND	ND	ND		ND	ND	ND	ND	ND
AL4	ND	ND	√	ND	√	√	ND	ND	ND	ND	ND	ND	ND		ND	ND	ND	ND	ND
AL5	ND	ND	√	√	√	√	ND	ND	ND	ND	ND	ND	ND		ND	ND	ND	ND	ND
AL8	ND	ND	ND	ND	√	√	ND	ND	ND	ND	ND	ND	ND		ND	ND	ND	ND	ND
AL9	ND	ND	√	√	ND	ND	ND	ND	ND	ND	ND	ND	ND		ND	ND	ND	ND	ND
AL10	ND	ND	√	√	ND	ND	ND	ND	ND	ND	ND	ND	ND		ND	ND	ND	ND	ND
AL11	ND	ND	ND	ND	√	√	ND	ND	ND	ND	ND	ND	ND		ND	ND	ND	ND	ND
AL12	ND	ND	ND	ND	√	√	ND	ND	ND	ND	ND	ND	ND		ND	ND	ND	ND	ND
AL13	ND	ND	ND	ND	√	√	ND	ND	ND	ND	ND	ND	ND		ND	ND	ND	ND	ND
AL14	ND	ND	ND	ND	√	√	ND	ND	ND	ND	ND	ND	ND		ND	ND	ND	ND	ND

√: detected; ND: not detected.

### Characterization of the PRO metabolites

**Figure 1:**
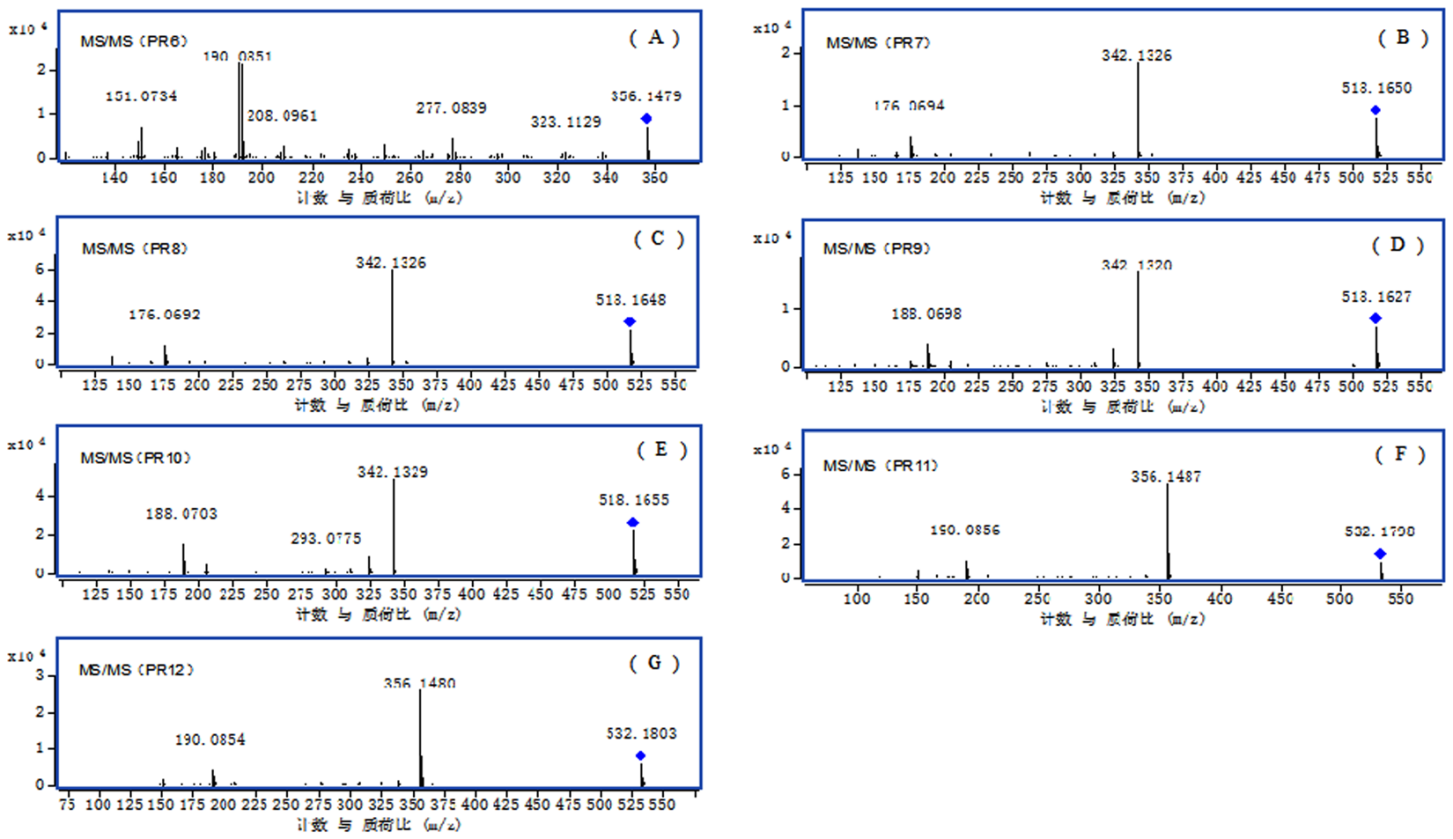
Accurate MS^2^ mass spectra PRO metabolites in rat *in vivo*: (**A**) PR6 (m/z 356); (**B**) PR7 (m/z 518); (**C**) PR8 (m/z 518); (**D**) PR9 (m/z 518); (**E**) PR10 (m/z 518); (**F**) PR11 (m/z 532); (**G**) PR12 (m/z 532).

A total of five PRO metabolites (PR1-PR5) were identified in rat liver S9 in our previous study^21^. In this study, three metabolites (PR1, PR2 and PR3) detected in rat liver S9 were found *in vivo* in addition to PR4 and PR5. The accurate MS^2^ spectra of seven other metabolites (PR6-PR12) detected *in vivo* are shown in Fig. 1. The retention times, predicted elemental compositions, observed and predicted masses, mass errors, and product ions for PRO metabolites are presented in Table 2. In all cases, the structures of metabolites andtheir product ions were rapidly and reliably characterized basedon the determined elemental composition and accurate MS^2^ spectra.

**Table 2:** The retention times (Rt), elemental compositions, observed masses and predicated masses, mass errors and product ions of PRO and its metabolites *in vivo*.

Compound	Rt (min)	Elemental composition	Observed mass	Predicated mass	Error (ppm)	Product ions (product ion error, ppm)
PRO	10.1	C_20_H_20_NO_5_^+^ ([M+H]^+^)	354.1331	354.1336	−1.41	336.1217(−3.89), 275.0682(−7.56), 206.0800(−5.7), 189.0767(−9.2), 188.0696(−5.37), 149.0586(−7.47)
PR1	9.0	C_20_H_22_NO_4_^+^ ([M+H]^+^)	356.1507	356.1492	4.08	322.1114(−5.79), 275.0756(−19.57), 247.0771(7.09), 206.0847(−16.98), 189.0793(4.63), 188.0720(7.46), 149.0616 (12.79)
PR2	7.1	C_19_H_20_NO_5_^+^ ([M+H]^+^)	342.1309	342.1336	7.91	308.0922(1.52), 263.0707(1.64), 235.0752(−0.67), 194.0815(1.71), 177.0757(−15.51), 176.0708 (1.11), 149.0575(−14.9)
PR3	8.2	C_19_H_20_NO_5_^+^ ([M+H]^+^)	342.1342	342.1336	1.76	275.0695(−2.81), 247.0760(2.62), 206.0790(−10.58), 189.0765(−10.26), 188.0705(−0.56), 137.0584 −9.6),
PR6	9.1	C_20_H_22_NO_4_^+^ ([M+H]^+^)	356.1479	356.1492	−3.8	338.1387(0.05), 323.1129(−7.17), 277.0839(−7.32), 208.0961(−3.48), 191.0924(−8.84), 190.0851(−6.11), 151.0734(−13.03), 149.0593(−2.74)
PR7	3.3	C_25_H_28_NO_11_^+^ ([M+H]^+^)	518.1650	518.1657	−1.33	342.1326(−2.93), 177.0757(−15.51), 176.0694(−8.03)
PR8	3.7	C_25_H_28_NO_11_^+^ ([M+H]^+^)	518.1648	518.1657	−1.72	342.1326(−2.93), 177.0764(−11.53), 176.0692(−8.03)
PR9	5.1	C_25_H_28_NO_11_^+^ ([M+H]^+^)	518.1627	518.1657	−5.78	342.1320(−4.69), 324.1226(−1.34), 189.0755(−15.58), 188.0698(−4.3)
PR10	5.9	C_25_H_28_NO_11_^+^ ([M+H]^+^)	518.1655	518.1657	−0.36	342.1329(−2.05), 324.1221(−2.89), 293.0775(−11.42), 189.0760(−12.92), 188.0703(−1.63)
PR11	7.5	C_26_H_30_NO_11_^+^ ([M+H]^+^)	532.1798	532.1813	−2.89	356.1487(−1.55), 191.0925(−8.31), 190.0856(−3.46), 151.0744(−6.37)
PR12	4.1	C_26_H_30_NO_11_^+^ ([M+H]^+^)	532.1803	532.1813	−1.95	356.1480(−3.52), 190.0854(−4.52), 151.0748(−3.71)

Metabolite PR6 eluted at a retention time of 9.1 min and showed the [M+H]^+^ ion at *m*/*z* 356.1479. According to our previous study^21^, the characteristic product ions of PRO at *m/z* 206.0800 and 149.0586 were formed by a retro-Diels-Alder (RDA) reaction from *m/z* 354.1331. As seen in Fig. 1, the product ions of PR6 at *m*/*z* 208.0961 and 149.0593 were also formed by an RDA reaction from *m*/*z* 356. The product ion at *m*/*z* 208.0961 showed a hydroxyl and a methoxyl linked to C-2 and C-3. The product ion at *m*/*z* 149.0593 suggested a methylenedioxy connected to C-9, C-10. The product ions at *m/z* 191.0924 and 190.0851 were formed by the loss of the OH radical and H_2_O, respectively, from *m/z* 208. Based on these observations, PR6 was tentatively identified as the ring-cleavage metabolite of PRO at the C2 or C3 bond.

Metabolites PR7, PR8, PR9 and PR10 had retention times of 3.3, 3.7, 5.1 and 5.9 min, respectively. They all showed the [M+H]^+^ ion at *m/z* 518.1650, 518.1648, 518.1627, 518.1655, respectively, which were 176 Da higher than PR2 (*m/z* 342.1309) or PR3 (*m*/*z* 342.1342), suggesting glucuronidation of PR2 or PR3. The MS^2^ spectra of PR7 and PR8 showed the same product ions at *m/z* 342.1326 and 176.0694. Metabolites PR7 and PR8 possessed the same characteristic product ion at *m/z* 176.0694 as PR2, indicating that they were formed by glucuronidation of PR2. The presence of a product ion at *m/z* 342.1326 also demonstrated that PR7 and PR8 were formed by glucuronidation of PR2 at the hydroxyl of C-2 or C-3. Therefore, PR7 and PR8 were tentatively identified as glucuronidation metabolite of PR2.

The MS^2^ spectra of PR9 and PR10 showed the same product ions at *m/z* 342.1320 and 188.0698 (Fig. 1). Metabolites PR9 and PR10 possessed the same characteristic product ion at *m/z* 188.0698 as PR3, indicating that they were formed by glucuronidation of PR3. The presence of product ion at *m/z* 342.1320 also demonstrated that PR9 and PR10 were formed by glucuronidation of PR3 at the hydroxyl of C-9 or C-10.

Metabolites PR11 and PR12 eluted at retention times of 7.5 min and 4.1 min, respectively. They both showed the [M+H]^+^ ion at *m/z* 532.1798 and 532.1803, respectively, which were 176 Da higher than PR6 (*m/z* 356.1479), suggesting glucuronidation of PR6. The MS^2^ spectra of PR11 and PR12 showed the same product ions at *m/z* 356.1487 and 190.0856. Metabolites PR11 and PR12 possessed the same characteristic production at *m/z* 190.0856 as PR6, indicating that they were formed by glucuronidation of PR6. The presence of production at *m/z* 356 also demonstrated that PR11 and PR12 were formed by glucuronidation of PR6 at the hydroxyl of C-2 or C-3.

### Biotransformation of ALL in plasma, urine and feces

The metabolites of ALL detected in the plasma, urine and feces of male and female rats after dosing are summarized in Table 1. No parent drug and ALL metabolite were detected in plasma at 3 h after intragastric administration of ALL in female and male rats. However, parent drug and five ALL metabolites—AL2, AL4, AL5, AL9, and AL10—were found in female rat urine at 0–24 h after intragastric administration of ALL. The accurateEIC of female raturine between 0 h and 24 h after oral administration of a single dose of ALL is shown in Fig. S2. (See supplemental material). In addition to the parent drug, four ALL metabolites—AL2, AL5, AL9, and AL10—observed in female rat urine were also observed in male rat urine at 0–24 h. As seen in Table 1, the parent drug and seven ALL metabolites—AL4, AL5, AL8, AL11, AL12, AL13, and AL14—were detected in both female and male rat feces. The accurateEIC of female rat feces between 0 h and 24 h after oral administration of a single dose of ALL is shown in Fig. S3. (See supplemental material). The results indicated that ALL was incompletely absorbed from the gastrointestinal tract and might be metabolized in the intestinal tract.

### Characterization of the ALL metabolites

**Figure 2:**
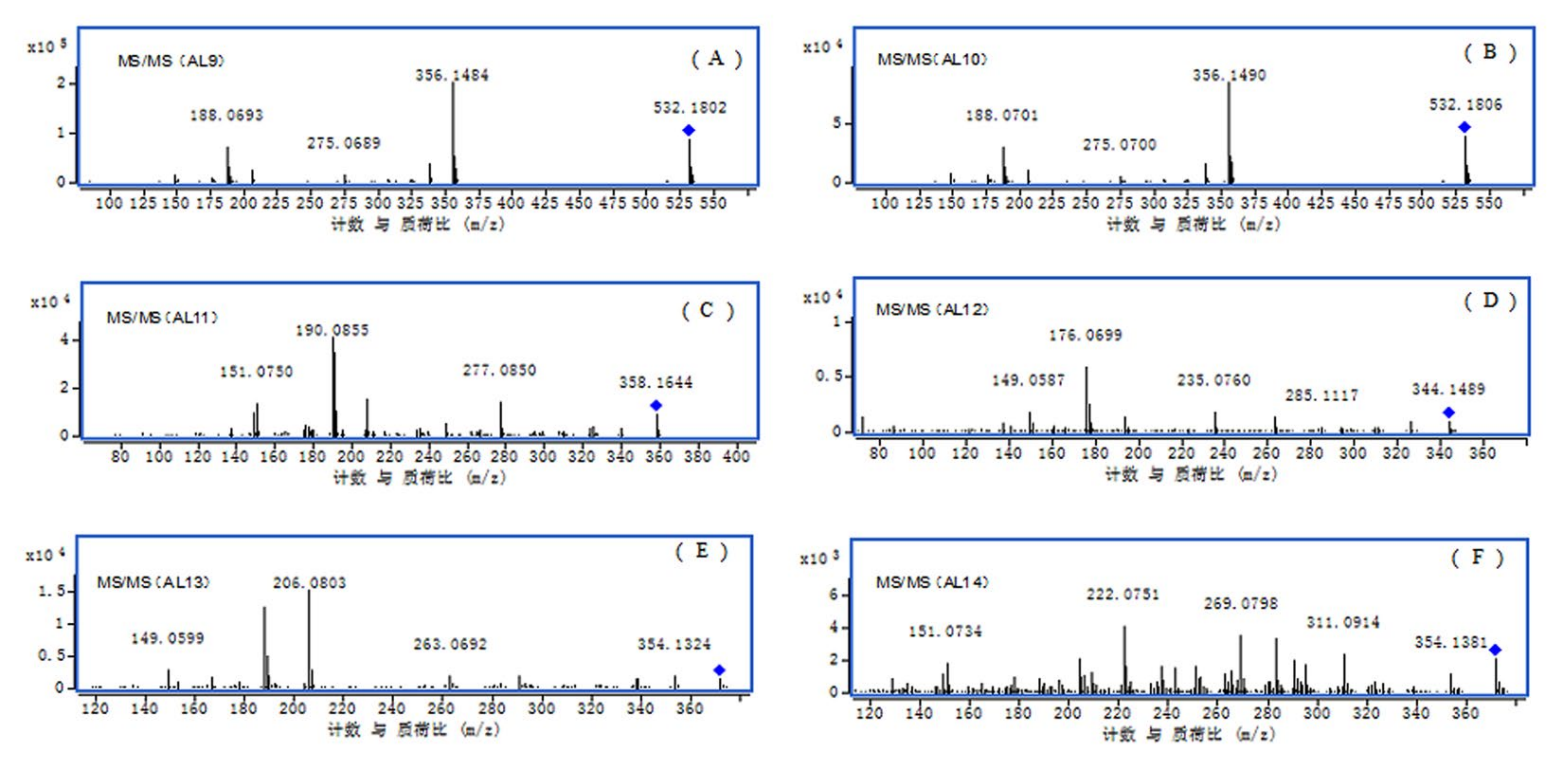
Accurate MS^2^ mass spectra ALL metabolites in rat *in vivo*: (**A**) AL9 (m/z 532); (**B**) AL10 (m/z 532); (**C**) AL11 (m/z 358); (**D**) AL12 (m/z 344); (**E**) AL13 (m/z 372); (**F**) AL14 (m/z 372).

A total of ten ALL metabolites were identified in rat *in vivo*. In addition to our previous reported metabolites AL2, AL4, AL5 and AL8^21^, six other metabolites (AL9-AL14) were also detected *in vivo*. The accurate MS^2^ spectra of these metabolites are shown in Fig. 2. The predicted elemental compositions, observed and predicted masses, mass errors, and product ions for ALL metabolites are presented in Table 3.

**Table 3:** The retention times (Rt), elemental compositions, observed masses and predicated masses, mass errors and product ions of ALL and its metabolites *in vivo* in rat.

Compound	Rt (min)	Elemental composition	Observed mass	Predicated mass	Error (ppm)	Product ions (product ion error, ppm)
ALL	10.4	C_21_H_24_NO_5_^+^ ([M+H]^+^)	370.1656	370.1649	1.9	352.1538(−1.52), 336.1231(0.2), 290.0933(−1.54), 206.0809(−1.32), 189.0774(−5.48), 188.0703(−1.63), 165.0906(−2.47)
AL2	9.6	C_21_H_26_NO_5_^+^ ([M+H]^+^)	372.1811	372.1805	1.48	354.1703(0.89), 322.1412(−8.00), 292.1083(−3.76), 208.0921(−22.59), 191.0904(−12.71), 190.0857(−2.94), 165.0897(−7.96)
AL4	8.4	C_20_H_24_NO_5_^+^ ([M+H]^+^)	358.1654	358.1649	1.4	340.1531(−3.64), 278.0930(−2.69), 194.0809(−1.4), 177.0759(−14.37), 176.0700(−3.46), 165.0893(−10.40)
AL5	8.8	C_21_H_22_NO_5_^+^ ([M+H]^+^)	356.1495	356.1492	0.71	338.1377(−2.92), 323.1127(−7.79), 275.0696(−2.45), 247.0735(−7.54), 206.0799(−6.19), 189.0766(−9.73), 188.0695(−5.91), 149.0587(−6.79)
AL8	3.4	C_19_H_22_NO_5_^+^ ([M+H]^+^)	344.1517	344.1492	7.14	311.1178(8.35), 263.0748(17.11), 235.0785(13.43), 194.0851(20.10), 177.0809(14.03), 176.0744(21.58), 149.0635(25.49)
AL9	4.9	C_26_H_30_NO_11_^+^ ([M+H]^+^)	532.1802	532.1813	−2.14	356.1484(−2.39), 338.1374(−3.81), 275.0689(−5.00), 189.0757(−14.52), 188.0693(−6.98), 149.0589(−5.44)
AL10	5.5	C_26_H_30_NO_11_^+^ ([M+H]^+^)	532.1806	532.1813	−1.39	356.1490(−0.70), 338.1389(0.64), 275.0700(−0.99), 206.0811(−0.34), 188.0701(−2.70), 189.0774(−5.48), 149.0589(−5.44)
AL11	6.3	C_20_H_24_NO_5_^+^ ([M+H]^+^)	358.1644	358.1649	−1.4	340.1558(4.32), 325.1315(1.98), 277.0850(−3.33), 208.0966(−1.06), 191.0931(−5.16), 190.0855(−3.99), 151.0750(−2.37)
AL12	5.1	C_19_H_22_NO_5_^+^ ([M+H]^+^)	344.1489	344.1492	−1.02	326.1392(1.59), 263.0688(5.61), 235.0730(−10.07), 194.0803(−4.50), 176.0699(−4.03), 177.0748(−20.33), 149.0587(−6.79),
AL13	6.5	C_20_H_22_NO_6_^+^ ([M+H]^+^)	372.1449	372.1442	1.98	354.1324(−3.40), 291.0654(0.74), 263.0692(−4.09), 189.0761(−12.32), 188.0696(−5.34)167.0696(−4.04), 149.0599(1.31)
AL14	8.0	C_20_H_22_NO_6_^+^ ([M+H]^+^)	372.1454	372.1442	3.33	354.1381(12.71)222.0751(−4.45), 205.0684(−26.82), 204.0645(−5.02), 151.0734(−13.03),

Metabolites AL9 and AL10 eluted at retention times of 4.9 min and 5.5 min, respectively. They both showed the [M+H]^+^ ion at *m*/*z* 532.1802 and 532.1806, respectively, which was 176 Da higher than AL5 (*m/z* 356.1492) or AL6 (*m/z* 356.1492), suggesting glucuronidation of AL5 or AL6. The MS^2^ spectra of AL9 and AL10 showed the same product ions at *m/z* 356.1484, 275.0689 and 188.0693 Metabolites AL9 and AL10 possessed the same characteristic product ion at *m/z* 188 as AL5 or AL6, indicating that they were formed by glucuronidation of AL5 or AL6. The presence of product ions at *m/z* 356, 275 also demonstrated that AL9 and AL10 were formed by glucuronidation of AL5 or AL6 at hydroxyl of C-9 or C-10. Therefore, AL9 and AL10 were tentatively identified as glucuronidation metabolite of AL5 or AL6.

Metabolite AL11 had a retention time of 6.3 min and showed [M+H]^+^ ion at *m/z* 358.1644, 12 Da lower than ALL, indicating that demethylation had occurred. As seen in Fig. 2, the MS^2^ spectra of AL11 exhibited a product ion at *m/z* 340.1558, which was formed by the loss of H_2_O from *m/z* 358.1644. The product ion at *m/z* 277.0850 lost NH_2_CH_3_ and CH_3_OH (observed 63.0708 Da, predicted 63.0684 Da) from *m/z* 340.1558. The product ions at *m/z* 208.0966and 151.0750 were also formed by an RDA reaction from *m/z* 358.1644. The product ion at *m/z* 208.0966 showed that a hydroxyl and a methoxyl were linked to C-2 and C-3. The product ion at *m/z* 151.0750 suggested that a hydroxyl and a methoxyl were connected to C-9 and C-10. The product ions at *m/z* 191.0931 and 190.0855 were formed by the loss of the OH radical and H_2_O, respectively, from *m/z* 208.0966. Therefore, AL11 was tentatively formed by ring cleavage at C-2 and C-3 and demethylation at C-9 or C-10 of ALL.

Metabolite AL12 had a retention time of 5.1 min and showed the [M+H]^+^ ion at *m*/*z* 344.1489, which was 12 Da lower than AL5 or AL6. Thus, AL12 was likely produced by the demethylation of AL5 or AL6. AL12 possessed the same [M+H]^+^ ion and product ions as AL8. Therefore, AL12 was tentatively identified as an isomer of AL8 (Huang *et al*.^21^).

Metabolites AL13 and AL14 had retention times of 6.5 min and 8.0 min, respectively. They both showed the [M+H]^+^ ion at *m/z* 372.1449 and 372.1454, which was 15.9957 Da and 15.9962 Da higher than AL5 and AL6, respectively. Therefore, AL13 and AL14 were tentatively identified as hydroxylated metabolites of AL5 or AL6.

As seen in Fig. 2, the MS^2^ spectra of AL13 generated a product ion at *m/z* 354.1324, which was formed by the loss of H_2_O from *m/z* 372.1449. The product ion at *m/z* 291.0654 was formed by the loss of NH_2_CH_3_ and CH_3_OH from *m/z* 354.1324. The product ion at *m/z* 263.0692 lost a CO from *m/z* 291.0654. The product ions at *m/z* 206.0803 and 167.0696 were also formed by an RDA reaction from *m/z* 372.1449. The product ion at *m/z* 206.0803 suggested methylenedioxy connected to C-2 and C-3. The product ion at *m/z* 167.0696 showed that a hydroxyl and a methoxyl were linked to C-9 and C-10 and that a hydroxyl was connected to C-11 or C-12. The product ions at *m/z* 188.0696 and 189.0761 lost an OH radical and H_2_O from *m/z* 206, respectively. Therefore, AL13 was tentatively identified as a hydroxylated metabolite of AL5 or AL6 at either C-11 or C-12.

The MS^2^ spectra of AL14 showed the product ion at *m/z* 354, which was formed by the loss of H_2_O from *m/z* 372.1454. The product ions at *m/z* 222.0751 and 151.0734 were also formed by an RDA reaction from *m/z* 372.1454. The product ion at *m/z* 222.0751 showed that the methylenedioxy was connected to C-2 and C-3 and that a hydroxyl was connected to eitherC1, C4, C5 or C6. The product ion at *m/z* 151.0734 showed that a hydroxyl and a methoxyl were linked to C-9 and C-10. The product ions at *m/z* 205.0684 and 204.0645 were formed by the loss of OH radical and H_2_O from *m/z* 222.0751, respectively. Therefore, AL14 was tentatively identified as the hydroxylated metabolite of AL5 or AL6 at either C-1, C-4, C-5 or C-6.

### Tissue distribution of PRO and ALL in rat tissues and cecal contents

As shown in Table 1, no ALL or metabolites were detected in rat cecal contents and tissues at 24 h and 48 h after intragastric administration of Plume Poppy Total Alkaloids for 3 weeks. Two PRO metabolites, PR2 and PR3, were found in rat cecal contents at 24 h after intragastric administration of Plume Poppy Total Alkaloids. In addition to PR2 and PR3, two other PRO metabolites, PR1 and PR6, were also observed in rat cecal contents at 48 h. However, no PRO metabolite was detected in heart, liver, spleen, lung or kidney 24 h or 48 h afterdosing.

After no PRO and ALL were found in tissues using HPLC-Q-TOF MS, the rat tissues and cecal contents were further analyzed by a sensitive HPLC-QqQ MS method for the determination of PRO and ALL. The distribution of PRO and ALL in rat tissue and cecal contents after intragastric administration of Plume Poppy Total Alkaloids for 3 weeks is shown in Table 4. The concentrations of ALL in cecal contents and tissues were less than 3 ng/g. The concentrations of PRO in cecal contents and tissues were less than 6.5 ng/g. The results showed that PRO and ALL are distributed in various tissues.

**Table 4:** The residue result of PRO and ALL in rat tissues (ng/g).

Tissues	ALL		PRO	
	24 h	48 h	24 h	48 h
liver	1.9	2.49	ND	6.18
heart	1.04	1.42	ND	ND
Spleen	0.99	2.73	1.28	5.42
Lung	1.93	0.96	0.9	2.08
Kidney	1.02	ND	1.23	ND
Cecal contents	1.77	2.94	1.05	6.05

### Effects on drug-metabolizing enzyme activity *in vivo*

#### Effect on CYP activity

**Figure 3:**
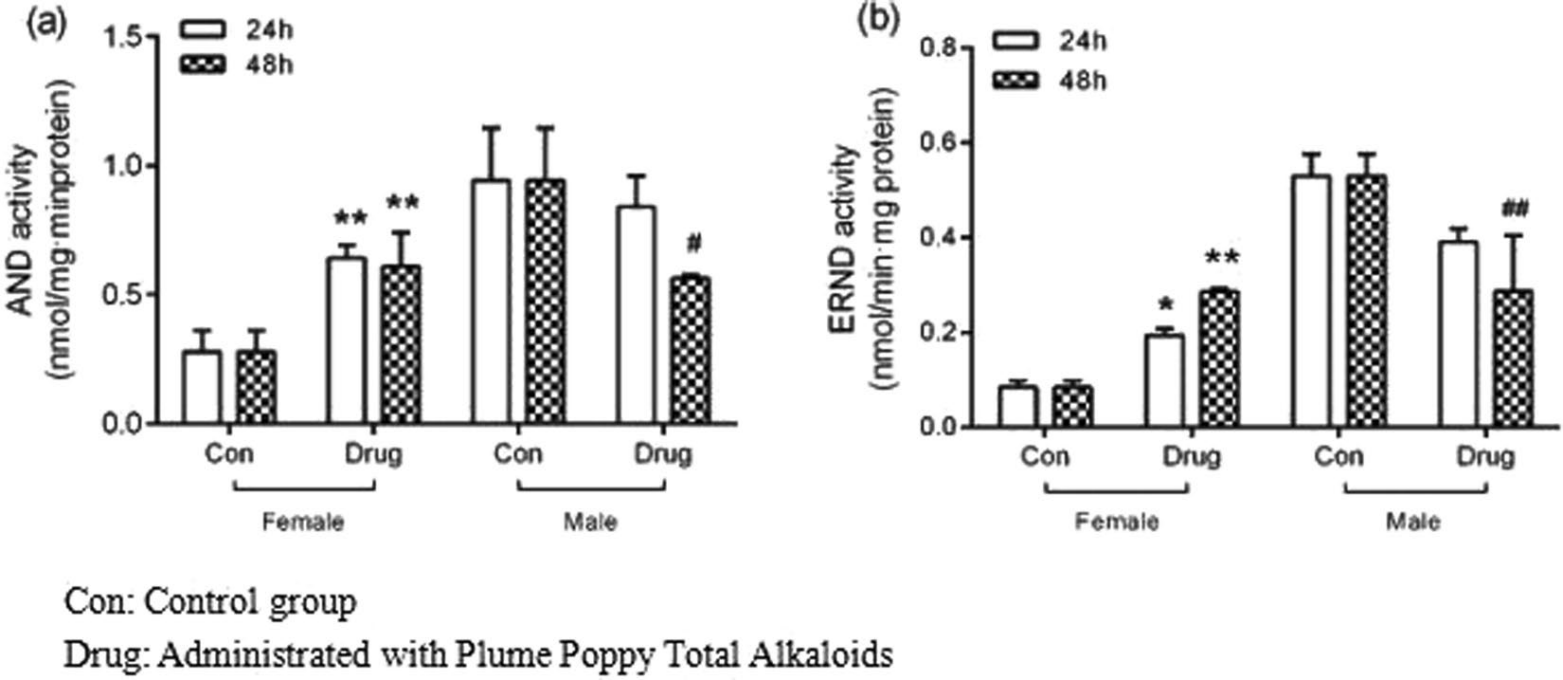
Assessment of *in vivo* AND (**a**) and ERND (**b**) activity in rats following intragastric administered with Plume Poppy Total Alkoloid. Each bar represents mean ± standard deviation of three observations 189.0761(−12.32), 188.0696(−5.34)167.0696 (−4.04), 149.0599(1.31) (n = 3). ^*/#^P < 0.05, ^**/##^P < 0.01 vs. respective control group of 24 h and 48 h, respectively.

As shown in Fig. 3a, the activity of AND was induced by Plume Poppy Total Alkaloids in female rats and was approximately two times higher than the control both 24 h and 48 h after last administration. However, significant inhibition of AND activity was found in male rats48 h post-dose. The AND activity of control male rat and treated male rat at 48 h was 0.94 and 0.56 nmol/min·mg protein, respectively.

As illustrated in Fig. 3b, ENRD activity was also induced in Plume Poppy Total Alkaloids-treated female rats, compared to the activity recorded in control, and the level reached statistical significance (P < 0.05). However, significant inhibition of AND activity was found in male rats after the last administration. The ERND activity of control male rats and treated male rats at 28 h was 0.53 and 0.39 nmol/min·mg protein, respectively.

#### Effect on NQO1 activity *in vivo*

**Figure 4:**
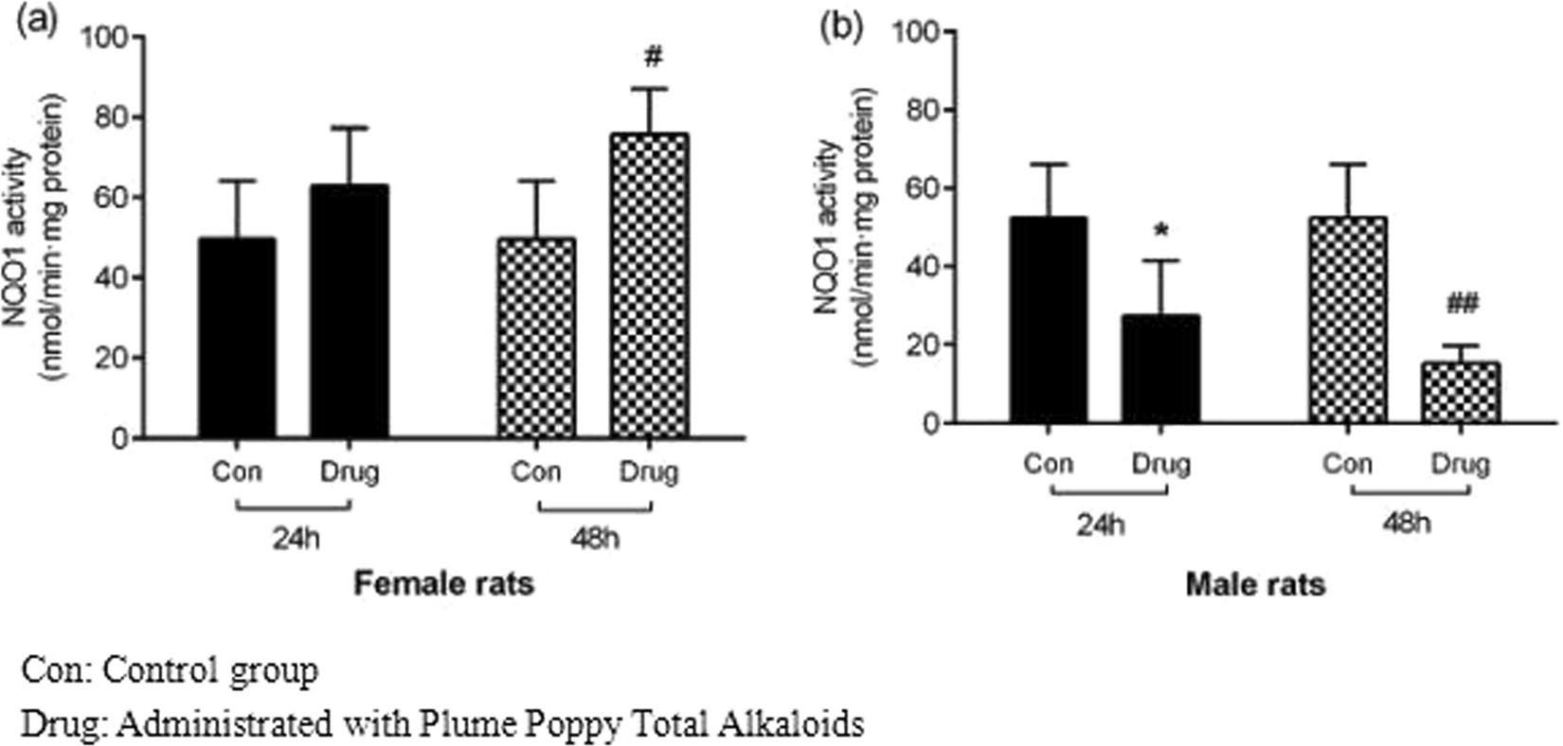
Assessment of *in vivo* NQO1 activity in female rats (**a**) and male rats (**b**) following intragastric administered with Plume Poppy Total Alkoloid.NADPH was used as electron donor. NOQ1 activity is presented as micromoles of 2, 6-dichlorophenolindophenol reduced per minute per milligram of protein. Each bar represents mean ± standard deviation of three observations (n = 3) ^*/#^P < 0.01, ^**/##^P < 0.05 vs. respective control group of female and male rat.

In the Plume Poppy Total Alkaloid-treated female rats, the enzyme activity of NQO1 at 24 h and 48 h increased by 21.23% and 35.49%, respectively, compared to control rats (Fig. 4a). In contrast, a decline inNQO1 activity was noted in male rats, and the enzyme activity of NQO1 was decreased at 24 h and 48 h by 47.55% and 70.85%, reaching statistical significance compared to the control (Fig. 4b).

## Discussion

In this study, the biotransformation of PRO and ALL were comprehensively investigated in rats after a single dose of PRO and ALL. Both compounds undergo extensive metabolism: ten PRO metabolites and ten ALL metabolites were detected in rats *in vivo*. In our previous work^21^, a total of five PRO metabolites (PR1-PR5) and eight ALL metabolites (AL1-AL8) were identified in rat liver S9 using an HPLC-QqTOF method. The ten PRO metabolites included four phase I metabolites (PR1, PR2, PR3, PR6) described by previous reports^17–19^ and six phase II glucuronidated metabolites (PR7-PR12) identified for the first time. However, no acetylated or sulfated metabolites of PRO were found in the current study^20^. In addition to four phase I metabolites (AL2, AL4, AL5, AL8) characterized in rat liver S9, two phase II glucuronidated metabolites (AL9- AL10) and four phase I metabolites (AL11-AL14) were also observed *in vivo* in the present study. Among these six metabolites (AL9-AL14), one of two phase II glucuronidated metabolites was characterized by a previous study^20^ and another four phase I metabolites (AL11-AL14) were found for the first time. The glucuronidated metabolite PR12 was found in the urine during the period of 0–24 h in male rat. However, PR12 was not observed in urine at 0–24 h after dosing in female rats. Metabolite AL4 was observed in urine 0–24 h after dosing in female ratsbut not male rats. Therefore, the biotransformation of PRO and ALL appeared to be qualitatively different between female and male rats.

**Figure 5:**
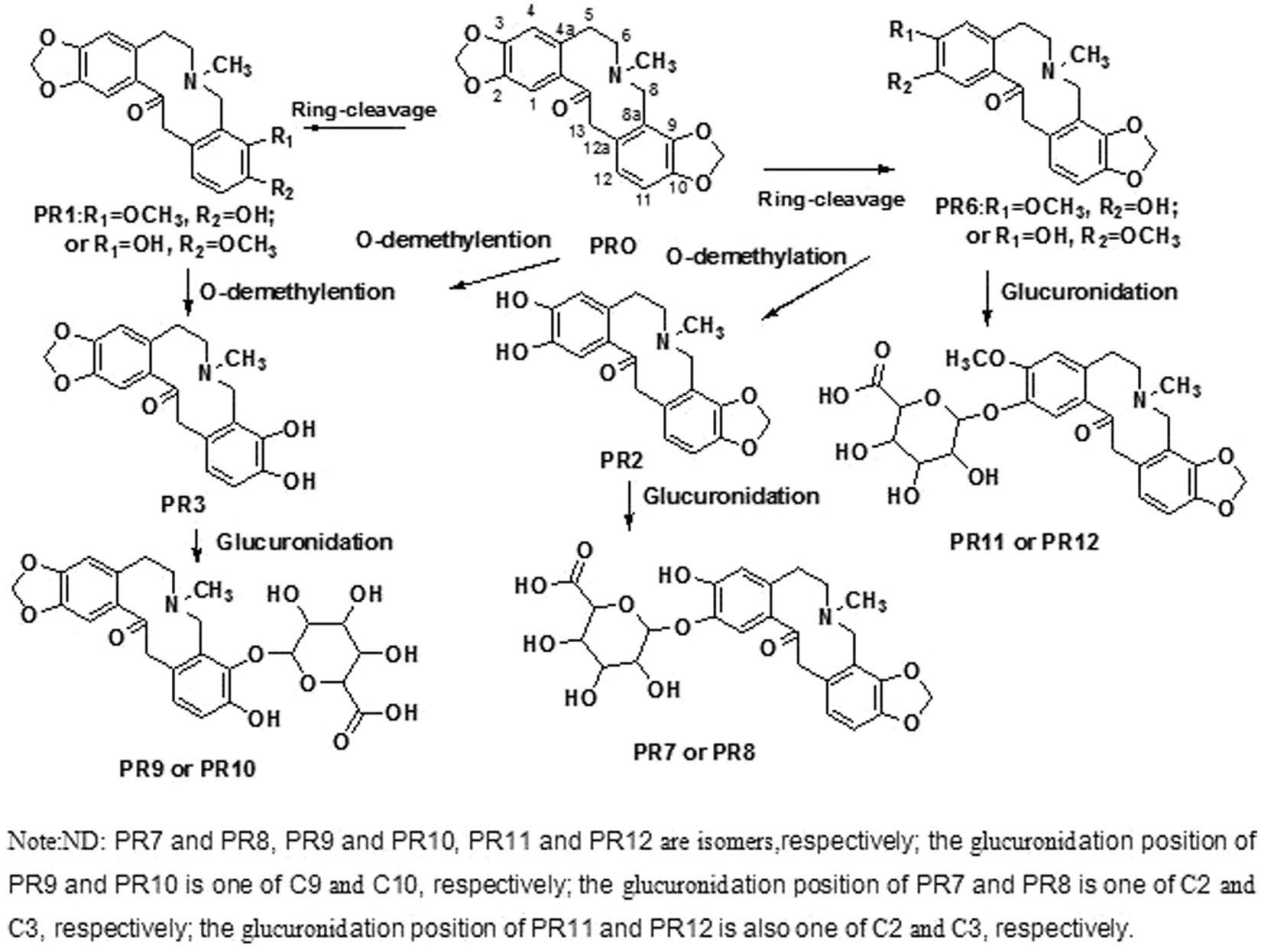
Proposed metabolic pathways of PRO in rat in *vivo*.

**Figure 6:**
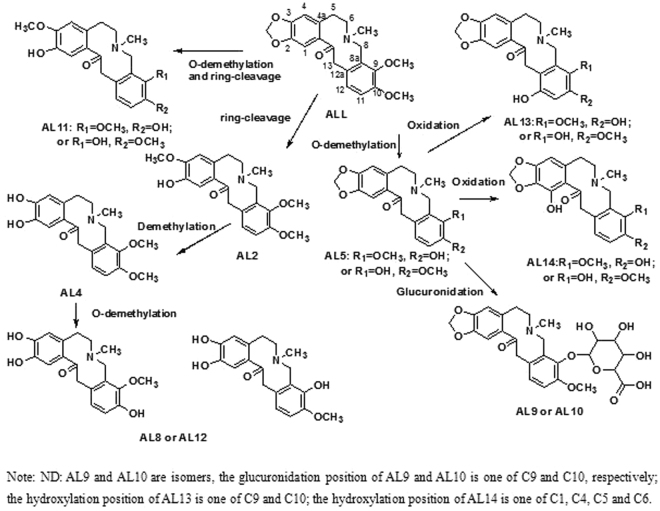
Proposed metabolic pathways of ALL in rat in *vivo*.

After oral administration of PRO or ALL in female and male rat, no parent drug or metabolites were detected in plasma at 3 h. Following oral administration of 2.0 g Rhizoma Corydalis Decumbentis extract per kg of body weight in Wistar rat, the T_max_ and T_1/2_ of PRO were 3.50 ± 0.55 h, 4.98 ± 1.64 h, respectively^14^. A previous pharmacokinetic study showed that the T_max_ and T_1/2_ of ALL were 30 min, 316.88 min, respectively^19^. The result indicated the absorption and excretion of PRO and ALL were fast in rat, in good agreement with previously reports on the pharmacokinetics of PRO and ALL. Based on the present results, the bioavailability of PRO and ALL is low; the two compounds undergo first-pass metabolism in the liver and reach very low concentrations in the plasma. Moreover, the metabolites PR2 and PR6 were detected in the feces in the study, while their glucuronidation metabolites PR7, PR8, PR11 and PR12 were only detected in urine. The metabolite AL5 was detected in feces, while the glucuronidation metabolites AL9 and AL10 were only detected in the urine. Therefore, we concluded that PRO and ALL may be metabolized in the intestinal tract and undergo enterohepatic circulation in rats. The demethylation of the 2, 3-methylenedioxy group was the main metabolic pathway for PRO and ALL *in vivo*. Metabolites AL4 and AL5 were both major metabolites in urine at 0–24 h after dosing in female rats and in the feces during the period of 0–24 h in female and male rats. The demethylations of the 2, 3-methylenedioxy group and 9, 10-vicinal methoxyl group were also the main metabolic pathways for ALL *in vivo*. The common metabolic pathways of PRO and ALL *in vivo* include ring cleavage, demethylation following ring cleavage and glucuronidation. Based on these results, the possible metabolic pathways of PRO and ALL *in vivo* were proposed as shown in Figs 5 and 6, respectively.

The isoquinoline alkaloids PRO and ALL are present in veterinary phytopreparations made from *Macleaya cordata* and used as a natural feed additive^6^. With increasing attention on food safety, the natural compound residue has become a main issues effecting human health. Thus, studying the metabolites and distribution in tissues of PRO and ALL after long-time treatment is an important issue. In the present study, no PRO and ALL or their metabolites were detected in the heart, liver, spleen, lung or kidney following intragastric administration with Plume Poppy Total Alkaloids for 3 weeks using the HPLC-QqTOF MS method. However, low concentrations (<10 ng/g) of PRO and ALL were determined by the HPLC-MS/MS method under some sample pretreatments. The major reason was that the sensitivity of the HPLC-QqTOF MS method HPLC-MS/MS was 20 ng/g and 0.5 ng/g for PRO and ALL, respectively. Our results also demonstrated that both compounds have lower residue concentrations in tissue. Therefore, we doubt that the low-concentration metabolites of PRO and ALL might be present in tissues. The lack of residue of phytopreparations in tissues may be explained by the fact that the investigations are seriously hampered by several factors, including extremely low parent drug levels and the metabolite concentrations of natural compound in tissues, as well as the insufficient sensitivity of analytical methods.

PRO and ALL are the biologically active substances in Traditional Chinese Medicine such as Yuanhu Zhitong tablet^26^. It is hence necessary to elucidate potential herb-drug interactions between PRO, ALL and other drugs so that they can be used safely and effectively. PRO and ALL have been also shown to inhibit several human CYP enzymes^23^. However, the effects of PRO and ALL on drug-metabolizing enzymes *in vivo* have not been assessed to date. The present study was designed to clarify the effect of Plume Poppy Total Alkaloid on ERND, ADM and NQO1 activity in female and male rats. ERND and AND were used as an indicator of CYP3A and CYPs catalytic activity, respectively^27,28^. Our group has demonstrated that NQO1 may be involved in isoquinoline alkaloid sanguinarine metabolism and decrease the toxicity of sanguinarine to cells^29^. The results showed that the activities of AND, ERND and NQO1 in female rats can be induced after treatment of rat with Plume Poppy Total Protopine, but inhibition activities were observed in male rats. As we known, many studies have demonstrated that hormone is the key hormonal factor that dictates sex differences in the expression of a large number of CYPs and other drug-metabolizing enzymes^30,31^. Pregnane X receptor and the constitutive androstane receptor are known to regulate the expression of drug-metabolizing enzymes and transporters^32^. A recently study has shown that PRO and ALL activated the pregnane X receptor *in vitro*^25^. Therefore, we suspected that the gender difference of PRO and ALL on drug-metabolizing enzymes may be relative to the difference in enzymes activities that were regulated by hormones. The difference mechanisms of these effects and the clinical significance of the herb-drug interactions will require further study in the future using molecular biology technology, such as RT-PCR and Western blotting.

In summary, the current study investigates the biotransformation of PRO and ALL in urine, plasma and feces following oral administration of PRO and ALL in rats for the first time. A total of ten PRO metabolites and ten ALL metabolites were also observed in rats *in vivo*. Among these, six PRO metabolites and five ALL metabolites were detected for the first time. The low concentration residue of PRO and ALL was detected in rat tissues following intragastric administration of Plume Poppy Total Alkaloid for 3 weeks. The ERND, ADM and NQO1 activities in female rats can be induced, while these activities were inhibited in male rats. There were qualitative and quantitative gender differences in the metabolism of PRO and ALL and effects on drug-metabolizing enzymes by PRO and ALL. These findings showed that herb-drug interaction should be taken into account when phytopreparations containing PRO and ALL are combined with other natural substances or drugs. These metabolism and tissue distribution results not only provide a theoretical basis for interpreting the pharmacokinetics of PRO and ALL but also provide a scientific basis for their food safety evaluation and application.

## Materials and Methods

### Chemicals

Protopine (PRO), Allocryptopine (ALL) and Plume Poppy Total Alkoloidsderived from *Macleaya cordata* (containing 35% PRO and 15% ALL) were obtained from Hunan Meikeda Co. (Changsha, China). HPLC-grade acetonitrile and formic acid were purchased from Merck (Darmstadt, Germany). Nicotinamide adenine dinucleotide phosphate hydrogen (NADPH) was purchased from Roche Chemical Co. (Beijing, China). Deionized water was purified using a Milli-Q system (Bedford, MA, USA). All other chemicals and reagents used were of the highest analytical grade.

### Animals and sample collection

Sprague-Dawley rats were obtained from the Hunan SLRC Laboratory Animal Center (Changsha, China). All experimental procedures were conducted in accordance with the guidelines of the FDA Good Laboratory Practice for Nonclinical Laboratory Studies^35^. All animal care and experimental protocols were conducts in accordance with the Guide for the Care and Use of Hunan Provincial Laboratory Animal Public Service Center (permit number SYXK 2010-0005) and approved by the Institutional animal care and use committee of Hunan Research Center for drug safety evaluation, Changsha, China. The animals were acclimatized for 1 week before the experiment.

#### Biotransformation

Twenty Sprague-Dawley rats (160–180 g, ten males and ten females) were individually housed in stainless steel metabolism cages. All rats were fasted for 12 h, but with access to water before dose administration. They were divided into two groups (five males and five females each group). One group was orally administered a 10 mg/kg dose of ALL in water with the dissolving aid of Tween-80. The other group was orally administered a 10 mg/kg dose of PRO. Heparinized blood samples of rats were collected 3 h after dosing. The collected blood samples were vortexed and centrifuged at 3000 rpm for 10 min at 4 °C. The urine and feces of rats were individually collected for 0–24 h post-dose. All samples from male and female rats from each group were pooled after collecting and stored at −20 °C.

#### Tissue distribution

Eighteen Sprague-Dawley rats (160–180 g, nine males and nine females) were divided into a drug group and control group. Twelve rats (six male and six female) were intragastrically administered with 5 mg/kg dose of Plume Poppy Total Alkaloids (containing 0.68 mg/kg dose of ALL and 1.83 mg/kg dose of PRO) in water with the dissolving aid of Tween-80 for 3 weeks. The rats in the control group (three male and three female) were intragastrically administered with an equal volume of water. Six rats (three male and three female) in the drug group and three rats in the control group were sacrificed 24 h after last administration, while the other rats were sacrificed at 48 h post-dose. Tissue samples (heart, liver, spleen, lung, kidney, and cecal contents) were collected. All samples from each group were pooled (male and female) after collecting at 24 h and 48 h post-dose because the amount of tissue was small.

### Sample Processing

#### Plasma and urine

100 µL of plasma was added with 400 µL ice-cold acetonitrile., mixed on a vortex mixer for 0.5 min. The sample was extracted with ultrasonic for 20 min and then centrifuged at 12, 000 rpm for 15 min. The supernatant was collected and filtered through 0.22 µm membrane filter. The clean supernatant was analyzed by HPLC-QqTOF MS for the identification of metabolites. 500 µL of urine was added with 500 µL ice-cold acetonitrile and the following procedures were carried out as plasma.

#### Feces

1.0 g of fecal was added to 5 mL hexane saturated acetonitrile-water (4:1, pH 5.0–5.5) and mixed on a vortex mixer for 0.5 min. The sample was extracted with ultrasonic for 30 min and then centrifuged at 10, 000 rpm for 15 min. The supernatant was collected and the residue was extracted again. The twice supernatants were pooled and was added to 5 mL n-hexane pretreated with acetonitrile. And the mixture was mixed on a vortex mixer for 0.5 min and centrifuged at 10, 000 rpm for 10 min. Then the layer liquid was collected and mixed with 0.3 g C18 and an appropriate amount of anhydrous sodium sulfate. The sample was mixed for 0.5 min and centrifuged at 10, 000 rpm for 5 min. The supernatant (5 mL) was collected and evaporated to dryness under nitrogen at 45 °C. The residue was reconstituted in 1 mL hexane saturated acetonitrile-water (4:1, pH 5.0–5.5) and analyzed by HPLC-QqTOF MS for the identification of metabolites.

#### Tissues

1.0 g of tissues (heart, liver, spleen, lung, kidney, and cecal contents) and 2 mL ultra-pure water were added to 50 mL of centrifugal tube. Then 15 mL of 0.2% formic acid alcohol-acetonitrile (90:10, v/v) solution was added and mixed on a vortex mixer for 1 min. The sample was extracted with ultrasonic for 30 min and then centrifuged at 7830 r/min for 10 min. The supernatant was collected and the residue was extracted again. The twice supernatants were pooled and was added to 5 mL n-hexane. And the tube was vortexed for 2 min and centrifuged at 10000 rpm for 2.0 min. The supernatant (5 mL) was collected and evaporated to dryness under nitrogen at 45 °C. The residue was reconstituted in 2 mL methanol, then mixed and centrifuged at 12, 000 rpm for 10 min. A portion of the supernatant was analyzed by HPLC-QqTOF MS for the identification of metabolites. Other portion was quantified by HPLC-MS/MS method.

### Sample Processing

#### Plasma and urine

One hundred microliters of plasma was added to 400 µL ice-cold acetonitrile and mixed in a vortex mixer for 0.5 min. The sample was extracted with ultrasonication for 20 min and centrifuged at 12, 000 rpm for 15 min. The supernatant was collected and filtered through a 0.22-µm membrane filter. The clean supernatant was analyzed by HPLC-QqTOF MS to identify metabolites. Five hundred microliters of urine was added 500 µL ice-cold acetonitrile, and the following procedures were followed as for plasma.

#### Feces

One gram of feces was added to 5 mL hexane-saturated acetonitrile-water (4:1, pH 5.0–5.5) and mixed in a vortex mixer for 0.5 min. The sample was extracted with ultrasonication for 30 min and centrifuged at 10, 000 rpm for 15 min. The supernatant was collected, and the residue was extracted again. The two supernatants were pooled and added to 5 mL n-hexane pretreated with acetonitrile. The mixture was mixed on a vortex mixer for 0.5 min and centrifuged at 10, 000 rpm for 10 min. Thelayer liquid was collected and mixed with 0.3 g C18 and an appropriate amount of anhydrous sodium sulfate. The sample was mixed for 0.5 min and centrifuged at 10, 000 rpm for 5 min. The supernatant (5 mL) was collected and evaporated to dryness under nitrogen at 45 °C. The residue was reconstituted in 1 mL hexane-saturated acetonitrile-water (4:1, pH 5.0–5.5) and analyzed by HPLC-QqTOF MS to identify metabolites.

#### Tissues

One gram of tissues (heart, liver, spleen, lung, kidney, and cecal contents) and 2 mL ultra-pure water were added to 50 mL in a centrifuge tube. Then, 15 mL of 0.2% formic acid alcohol-acetonitrile (90:10, v/v) solution was added and mixed in a vortex mixer for 1 min. The sample was extracted with ultrasonication for 30 min and centrifuged at 7830 r/min for 10 min. The supernatant was collected, and the residue was extracted again. The two supernatants were pooled and added to 5 mL n-hexane. The tube was vortexed for 2 min and centrifuged at 10, 000 rpm for 2.0 min. The supernatant (5 mL) was collected and evaporated to dryness under nitrogen at 45 °C. The residue was reconstituted in 2 mL methanol, mixed and centrifuged at 12, 000 rpm for 10 min. A portion of the supernatant was analyzed by HPLC-QqTOF MS to identify metabolites. The other portion was quantified by HPLC-MS/MS.

### Instruments and analytical conditions

#### HPLC-QqTOF MS

The HPLC analysis was performed using a Agilent 1290 HPLC system consisting of a autosampler, a rapid resolution binary pump, vacuums degasser, and thermostatically controlled column compartment. The separation was achieved on a Hypersil GOLD column (150 mm × 2.1 mm I.D.; particle size 5 μm). The mobile phase consisted of mobile phase A (0.1% formic acid) and mobile phase B (acetonitrile). The LC condition was optimized as followed: 10% B (0–5 min), 10%-90% B (5–20 min), 90% B (20–25 min), 10% B (25–30 min). The injection volume was 5 μL and the flow rate was 0.3 mL/min.

The QqTOF-MS system consisted of 6530 Q-TOF-MS accurate-mass spectrometer equipped with an electrospray ionization (ESI) source. It was operated in the positive mode. Nitrogen was used as the nebulizing gas at a flow rate of 9 L/min. The sheath gas temperature was maintained at 350 °C and the flow rate was 11 L/min. The nebulizer voltage, capillary voltage and nozzle voltage were set at 35 psig, 4.0 kv and 1kv, respectively. The isolation window was set at 1 amu. The instrument performed the internal mass calibration automatically by an automated calibrate delivery system. The calibrating solution contained the internal reference masses at m/z 121.0508 and 922.0098 in positive ion mode. All the data acquisition was controlled by Agilent Mass Hunter software (version B.01.03 Build 1.3.157.0 2).

#### HPLC-MS/MS

For quantification of PRO and ALL in tissues, analysis was performed on an Agilent series 1290 Infinity HPLC instrument coupled to an Agilent 6460 QQQ mass spectrometer (Agilent Technologies, Santa Clara, CA, USA). The chromatographic separation was performed on a 1.8 μm Agilent Zorbax SB-C_18_ (2.1 × 50 mm) column. The mobile phase consisted of mobile phase A (0.1% formic acid) and mobile phase B (acetonitrile). The linear gradient was as follows: 0–4 min, 15–17%B; 4–6 min, 17–35%B; 6–8 min, 35%B. The flow rate was 0.3 mL/min, and the injection volume was 2 μl.

Mass spectrometric detection operated in positive ESI mode using multiple reactions monitoring (MRM) mode. The operating parameters were as follows: capillary voltage, 3500 V; EM voltage, 200 V; dry gas (N_2_) flow rate, 12 L/min; ion source temperature, 350 °C; nebulizer, 25 psi. The quantitative transitions, collision energy (CE) and cone-voltage for PRO were 354.1 > 189.1, 34 eV and 110 V, respectively. The quantitative transitions, collision energy (CE) and cone-voltage for ALL were 370.2 > 188.1, 30 eV and 108 V, respectively.

Using optimal conditions, the retention times of PRO and ALL were 3.5 min and 4.4 min, respectively. The proposed method was validated by evaluating specificity, linearity, recovery, accuracy, precision, LOD and LOQ according to Commission Decision 2002/657/EC. The estimated limit of quantification for PRO and ALL in tissues using this method was 0.5 ng/g. The mean intra- and inter-day assay accuracies fell within 81.3–108.4% and 86.2–104.8% m, respectively. The mean intra- and inter-day precisions were 11.2 and 12.5% (RSD < 15%), respectively. The proposed method has proved to be suitable for accurate quantitative determination of PRO and ALL for tissue distribution analysis.

### Assay for drug-metabolizing enzymatic activity

Microsomes and cytosol of animal liver were prepared according to our previously published method^33^. Protein content was determined using the BCA protein assay kit, with bovine serum albumin as standard.

#### Assay for CYP activity

The effects on CYP enzymatic activity by PRO and ALL was evaluated as erythromycinN-demethylase (ERND) and aminopyrine N-demethylase (AMD). The method for the N-demethylase activity assay followed the work published by Azizi^34^ and was optimized for the current study. Briefly, an appropriate amount of microsomal suspension was added to a cuvette containing 250 μL of 0.1 M potassium phosphate buffer (PH = 7.4), 0.4 mM erythromycin or 0.8 mM aminopyrine as the substrate. After preincubation at 37 °C for 3 min in a water bath, the reaction was started by the addition of 0.5 mM NADPH (final concentration), and the tubes were further incubated for 20 min at 37 °C and stopped by the addition of 12.5% trichloroacetic acid. The supernatant was heated with Nash reagent at 60 °C for 10 min in a water bath and cooled. Subsequently, 150 μL from each tube was aliquoted into a 96-microtiter-well plate, and the absorbance at 405 nm was measured using a CHAMELEON ™ multitechnology plate reader. Both erythromycin and aminopyrine underwent N-demethylation to liberate formaldehyde. ERND and AND activities were expressed as nanomoles of HCHO reduced per minute per milligram of protein.

#### Assay for NAD (P)H quinone oxidoreductase (NQO1) activity

NQO1 specific activity was measured in treated rat liver cytosolic fraction by previously described procedures^35^, modified and adapted to a microplate reader^36^. The reaction mixture contained 50 mM Tris-HCl (pH 7.4), 40 μM DCPIP and 0.2 mM NADH in a final volume of 210 μL. Ten microliters of sample were pipetted into microplate wells, and the reaction was started by the addition of 200 μL of reaction mixture. Samples were measured with or without 40 μM dicoumarol and dissolved in 0.15% NaOH. Any change in absorbance was monitored at 620 nm, and NQO1 activity is defined as the dicumarol-inhibitable reduction of 2, 6-dichlorophenol-indophenol, calculated using the extinction coefficient for DCPIP (ε = 21 mM^−1^ cm^−1^) and expressed as nanomoles of DCPIP reduced per minute per milligram of protein.

#### Statistical Analysis

All results were analyzed by one-way analysis of variance (ANOVA) and Dunnett’s multiple comparison tests with Graphpad Prism6.0. Differences between means were determined at the p < 0.05 or p < 0.01 levels for all analyses and indicated with * and **, respectively. Data are represented as mean ± S.D. of at least three replicate experiments.

## Electronic supplementary material

Supplemental Material
