# Supplementary material for: Biotransformation and tissue distribution of protopine and allocryptopine and effects of Plume Poppy Total Alkaloid on liver drug-metabolizing enzymes
Source: Sci Rep. 2018 Jan 11;8:537. doi: 10.1038/s41598-017-18816-7 (PMC5765031; doi:10.1038/s41598-017-18816-7)
Supplement: Supplementary file 1 — Supplemental Material [file 41598_2017_18816_MOESM1_ESM.doc]

**Biotransformation and tissue distribution of protopine and allocryptopine and effects of Plume Poppy Total Alkaloid on liver drug-metabolizing enzymes**

Ya-Jun Huang, Pi Cheng, Zhuo-Yi Zhang, Shi-Jie Tian, Zhi-Liang Sun, Jian-Guo, Zeng, Zhao-Ying Liu


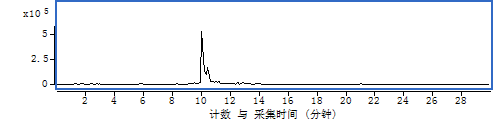


（A）

+EIC（354.1336）

PRO


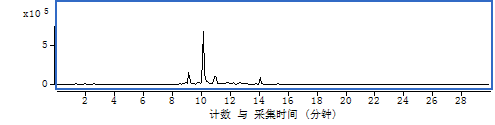


（B）

+EIC（356.1492）

PR6


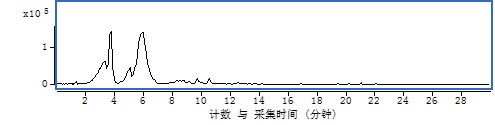


PR8

PR7

PR9

（C）

+EIC（518.1657）

PR10


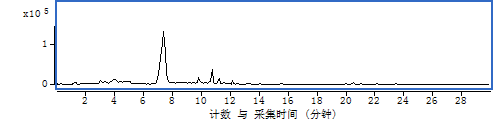


+EIC（532.1813）

（D）

PR11

**Supplementary Figure S1**. The accurate EIC of PRO and it’s metabolites in female SD rat 0-24 h urine; (A): PRO; (B): PR6 (*m/z* 356); (C):PR7, PR8, PR9, PR10 (*m/z* 518); (D): PR11 (*m/z* 532)


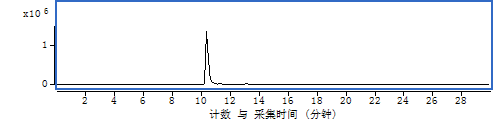


+EIC（370.1649）

（A）

ALL


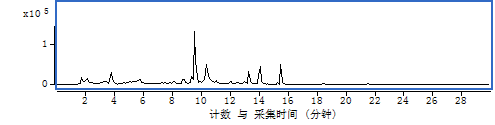


+EIC（372.1805）

AL2

（B）


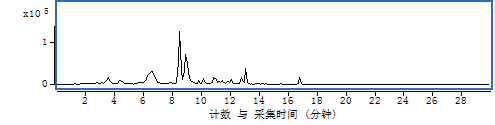


（C）

+EIC（358.1649）

AL4


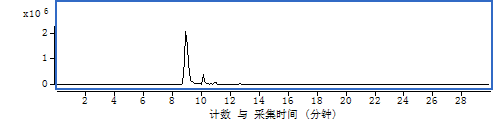


AL5

（D）

+EIC（356.1492）


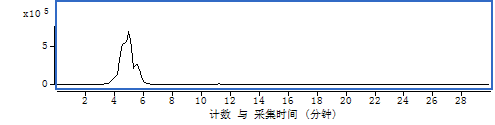


（E）

+EIC（532.1813）

AL9

AL10

**Supplementary Figure S2.** The accurate EIC of ALL metabolites in female SD rat urine at 0-24 h: (A) ALL (*m/z* 370); (B) AL1 (*m/z* 372); (C) AL4 (*m/z* 358);(D) AL5 (*m/z* 356); (E) AL9 and AL10 (*m/z* 532)


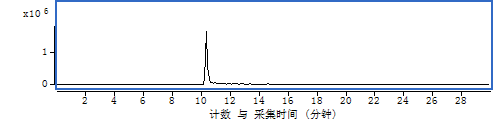


ALL

（A）

+EIC（370.1649）


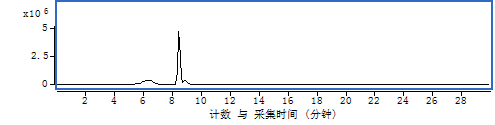


+EIC（358.1649）

（B）

AL11

AL4


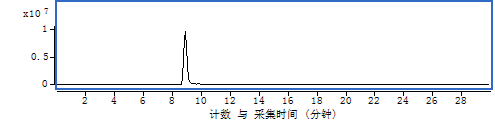


+EIC（356.1492）

（C）

AL5


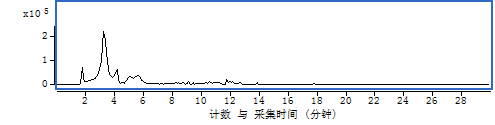


+EIC（344.1492）

（D）

AL12

AL8


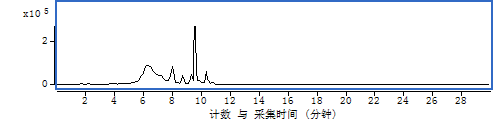


（E）

+EIC（372.1442）

AL14

AL13

**Supplementary Figure S3.** The accurate EIC of ALL metabolites in female SD rat feces at 0-24 h: (A)ALL (*m/z* 370); (B) AL4 (*m/z* 358) and AL11 (*m/z* 358); (C)AL5 (*m/z* 356);(D) AL8 (*m/z* 344) and AL12 (*m/z* 344);(E) AL13 (*m/z* 372) and AL14 (*m/z* 372)
